# Empowerment for behaviour change through social connections: a qualitative exploration of women’s preferences in preconception health promotion in the state of Victoria, Australia

**DOI:** 10.1186/s12889-022-14028-5

**Published:** 2022-08-30

**Authors:** Ruth Walker, Sara Quong, Patrick Olivier, Ling Wu, Jue Xie, Jacqueline Boyle

**Affiliations:** 1 Health and Social Care Unit, Monash University, 553 St Kilda Road, Melbourne, VIC 3004 Australia; 2 Eastern Health Clinical School, Faculty of Medicine, Nursing and health Sciences, Monash University, Level 2, 5 Arnold Street, Box Hill, VIC 3128 Australia; 3 Action Lab, Faculty of Information Technology, Monash University, Wellington Road, Clayton, VIC 3168 Australia

**Keywords:** Preconception, Lifestyle, Health, Behaviour change

## Abstract

**Background:**

Health behaviours in the preconception period have the potential to impact on fertility and pregnancy outcomes, and the health of all women regardless of pregnancy intention. Public awareness of this is low and interventions that promote behaviour change have not been integrated into real-world settings. Aims were to explore women’s understandings of health and health behaviours and what supports are important to promote behaviour change in the preconception period.

**Methods:**

This qualitative study is the first phase of a broader co-design project set in the state of Victoria, Australia. Over 3 months, a series of in-depth interviews were conducted with female participants who were intending to become pregnant in the next 2 years (*n* = 6) and participants who were not intending to become pregnant in the next 2 years (*n* = 6). Community advisors (*n* = 8) aged 18-45 years provided feedback throughout the process. Coding of transcripts from interviews and meetings was undertaken by two researchers before a deductive process identified themes mapped to the COM-B framework.

**Results:**

Nine themes and eight sub-themes were identified. Participants had a holistic view of health with nutrition, physical activity and sleep being most valued. Social connections were considered as being important for overall health and wellbeing and for promoting health behaviours. The only difference between groups was that pregnancy was an additional motivator for women who were planning to become pregnant in the next 2 years. A range of health information is available from health professionals and other sources. Unlimited access to information was empowering but sometimes overwhelming. Being listened to and shared experiences were aspects of social connections that validated participants and guided them in their decision-making.

**Conclusions:**

Women valued their health and had a holistic view that includes physical, mental and social dimensions. Women viewed social connections with others as an opportunity to be listened to and to gain support that empowers behaviour change. Future interventions to promote behaviour change in preconception women should consider the importance all women placed on social connections and leverage off existing resources to connect women.

**Supplementary Information:**

The online version contains supplementary material available at 10.1186/s12889-022-14028-5.

## Background

Women’s health in the preconception period impacts on fertility, pregnancy outcomes, infant health and the future health and wellbeing of mothers and their children [1–3]. The preconception period spans from the onset of menarche to menopause [2]. Preconception care tends to focus on women who are actively planning to become pregnant and the critical weeks around conception. A public health view of preconception considers the broader lifecourse approach where health and health behaviours years or even decades prior to conception have the potential to impact on pregnancy outcomes and all women’s health, including women who are not planning a pregnancy [4].

Community awareness of the importance of preconception health is low, including an understanding of the long term reproductive health impact of health behaviours and conditions well before a pregnancy [5]. Many women only seek preconception care if they do not become pregnant after trying to conceive [6]. An example of this is seen in the increasing prevalence of unhealthy weight in women of reproductive age, with almost 30% of women starting pregnancy overweight or with obesity [7]. Gaining excess weight may be frustrating for some women as they manage their general health [8] and there tends to be a limited understanding of the negative impact of overweight and obesity on reproductive health and fertility [9]. Rates of overweight and obesity are rising most rapidly in women during early adulthood [10], highlighting the importance of weight gain prevention in the first instance [11]. Women should ideally be supported to achieve a healthy weight in the months or even years before conception to benefit not only their own health but future fertility, pregnancy outcomes and health of the next generation [12, 13]. Importantly, aiming for healthy weight regardless of current pregnancy plans can improve health for women and their babies where pregnancy is unplanned and also improve overall health and well-being of women who may never have a pregnancy [2, 14].

From a lifecourse and market segmentation perspective, women may be classified as pregnancy ‘intenders’ (critical weeks around conception or actively planning to become pregnant) and pregnancy ‘non-intenders’ (when it is possible for a woman to become pregnant and her health behaviours have the potential to impact on pregnancy outcomes but she is not planning a pregnancy) [15]. A plethora of targeted interventions for women who are pregnancy intenders have been reported, including those promoting nutrition and prenatal supplementation [16–19], substance use [12, 17], and weight [17, 20], to name a few. Outcome measures (e.g., increased knowledge, behaviour change, health) for preconception interventions have varied, with most reporting on changes in knowledge rather than behaviour change [21]. Women identifying as non-intenders were likely to have been included in some of these interventions, however, interventions specifically targeting non-intenders are generally harder to identify as the use of terms such as preconception health and healthy pregnancy are not always used. The Show Your Love Today campaign in the United States targets ‘all people of a reproductive age’ to achieve optimal health for them and the children they may have [15, 22]. To the authors’ knowledge, this campaign has not been formally evaluated. When considering the reported lack of knowledge regarding key preconception health issues [23, 24], it seems that public health messages have not engaged women either as pregnancy non-intenders or intenders regarding the importance of preconception health and care. The aims of this study were to explore contemporary women’s understandings of health and health behaviours and what supports are important to promote behaviour change. This research is part of the first phase of a broader co-design project that aims to develop digital health resources that support women, both intending and not intending to become pregnant to optimise lifestyle behaviours in the preconception period.

## Methods

### Study design

A cycle of planning, data collection, analyses and reflection was undertaken to deeply engage with participants and understand their lived experiences in relation to health behaviours and supports for behaviour change. Data were qualitatively analysed for common and divergent perspectives and then synthesised into themes. Two community advisory committees guided this research from the outset. Ethics approval was obtained from the Monash University Human Research Ethics Committee (MUHREC reference: 28204). All research was conducted in accordance with relevant guidelines and regulations of the Monash University Human Research Ethics Committee.

### Setting, participants and community advisors

All but one community advisor lived in Victoria, Australia and were aged 18-45 years. One community advisor lived in New South Wales. The researchers agreed that having a community advisor from another Australian state would not impact on the purpose and role of the community advisor groups. The researchers deemed it important to involve community advisors from the outset of this research project in accord with Australia’s National Health Medical Research Council’s guidance for consumer and community involvement in health and medical research [25]. The main role of the community advisors was to engage with the researchers and provide feedback and advice on the evolution of the research plan. Participants lived in Victoria, Australia and were aged 18-45 years. Community advisors and participants needed to identify as being either i) an intender who was planning a pregnancy in the next 2 years or who had a child in the past 2 years and could recall their experience, or ii) a non-intender who was not planning a pregnancy in the next 2 years, with or without children already [15]. Those who could not speak English were excluded due to the unavailability of a translation service.

### Recruitment and consent

Participants and community advisors were recruited via advertisements sent to a range of women’s health groups in metropolitan Melbourne and regional Victoria, Australia and via Facebook and Twitter. Community advisors were recruited in February and March 2021. They responded to the study email with an expression of interest and were screened in a five-minute phone conversation with one of the researchers (RW; a female postdoctoral researcher with expertise in preconception and pregnancy health, dietetics and qualitative research methods). The recruitment of community advisors and the first community advisory committee meeting were completed before participant recruitment began. Community advisors consented to participate via an online consent form (via Qualtrics) after reading the Explanatory Statement, and were given a $40 gift voucher for every hour of input.

Potential participants were recruited in April and May 2021. They also expressed their interest via email. They were then sent an Explanatory Statement and underwent a 5 min screening interview (RW). Upon eligibility, participants consented to participate via an online consent form and were given a $40 gift voucher for every hour of input.

### Data collection

The community advisors were divided into two committees, intenders and non-intenders. Community advisors participated in four meetings each between March and October 2021 (four for intenders and four for non-intenders, facilitated by RW with oversight of JB, a female senior academic-clinician with expertise in obstetrics-gynaecology and equity in healthcare). These meetings were conducted online via Zoom due to COVID-19 lockdown restrictions in Victoria. The objectives of the first meeting (March 2021) were to i) introduce community advisors to the research team, and ii) introduce the overall study including aims and the study design. There was time for feedback at the end (this meeting was not audio-recorded). The objective of the second meeting (June 2021) was to i) introduce our participants (de-identified with participant codes) and to get feedback regarding the plans for the first in-depth interview. The objectives of the third (August/September 2021) and fourth (October 2021) meetings were to i) provide feedback regarding results so far, and ii) seek community advisors’ opinions of next steps in the research.

Three in-depth online interviews were conducted with each participant between June and September 2021. These interviews were designed to engage with women, and to understand their lived experiences in relation to their health and desired supports for healthy lifestyle behaviours. A range of activities were designed to assist with engagement, including visual prompts, visual diaries and case studies. Interview one (June/July 2021) focused on women’s perceptions of health and health behaviours. Interview two (July/August 2021) was informed by the first interview and focused on social connections for health. Interview three (August/September 2021) was informed by the previous two interviews and focused on digital health information and supports. Each interview was designed to go for approximately 1 h and was led by a member of the research team (RW or SQ, a female research assistant and dietitian with expertise in qualitative research methods). Again, all interviews were conducted online via Zoom due to COVID-19 lockdown restrictions in Victoria.

### Data analyses

The larger body of co-design research, of which this study is phase one, is ultimately a behaviour change intervention to empower women to adopt and maintain healthy lifestyle behaviours that optimise their preconception health and overall health and wellbeing. Behaviour change interventions, ‘a coordinated set of activities designed to change specified behaviour patterns,’ should be guided by theory [26]. While a range of behaviour change theories exist, the COM-B system was selected to support data analyses. In the COM-B system, an individual’s capability (psychological and physical capacity, including knowledge and skills), opportunity (factors external to an individual that make a behaviour possible, including physical environment and cultural milieu) and motivation (brain processes that energise and direct behaviour, including goal-setting, habits, emotional responses, informed decision-making) interact to elicit a behaviour [26]. The COM-B system was selected as a framework for the analyses because it acknowledges a range of intrinsic and extrinsic factors that may drive an individual’s behaviours and has been widely used as a framework for understanding women’s health behaviours [27–29].

Data collection and data analysis occurred concurrently. This was because data collected in previous interviews informed the interviews that followed. Two researchers (SQ, RW) co-coded a subset of the transcripts for interview one to develop the initial coding framework. Next, they independently coded the remaining transcripts from this first interview, with cross-checking for consistency. Between interview one and interview two, the research team (SQ, RW, LW, JX) developed questions and activities for the second interview. The same process of co-coding and individual coding was applied for interview two transcripts, with the initial coding framework being used as a basis and new codes being added. Again, between interview two and three, the research team developed questions and activities for interview three. Finally, a sub-set of in-depth interview three transcripts was co-coded and more codes were added to the initial coding framework before the remaining transcripts were coded. The transcripts from the community advisory committee meetings were each co-coded using the same coding framework. NVivo software supported the analyses.

A deductive process was adopted in the development of themes, whereby the codes were mapped to three focus areas of the in-depth interviews (perceptions of health and health behaviours, social connection, digital health information and support) and to the three components of the COM-B system (capability, opportunity, motivation). Two researchers (SQ, RW) developed summary statements for each intersection between the focus areas and the COM-B system. Themes and sub-themes were derived from the summary statements. This process was scrutinised by other members of the research team before the results were finalised.

## Results

Nine community advisors were recruited, three intenders and six non-intenders. A community advisor in the intender group attended the first meeting only (no reason for dropping out provided), leaving two advisors in the remaining meetings for the intender group. Six community advisors in the non-intender group attended meetings one to three, and four attended the fourth meeting (reasons for non-attendance were being caught in traffic and having to work late). Fourteen participants were recruited, six intenders and eight non-intenders. After providing consent via the online form, two of the non-intenders did not attend their first scheduled interview. This left six intenders, two of whom became pregnant during the study period, and six non-intenders who participated in all three of the interviews between June and September 2021 (Table 1).

**Table 1:** Community advisor and participant characteristics

	n (%)
**Community advisors (*n*** **= 8)**^a^	
Indenter	2 (25.0%)
Non-intender	6 (75.0%)
Age, median (IQR)	29 (11)
CALD	4 (44.4%)
Living with a disability^b^	2 (22.2%)
University educated	8 (88.8%)
**Participant (*n*** **= 12)**^c^	
Intender	6 (50.0%)
Non-intender	6 (50.0%)
Age, median (IQR)	31.5 (6)
CALD	3 (25.0%)
Living with a disability^b^	5 (41.7%)
University educated	10 (83.3%)

^a^Community advisor who only participated in the first session not included

^b^Post traumatic stress, hearing impairment, mental health, severe endometriosis, attention deficit and hyperactive disorder

^c^The two participants who did not engage in the first session are not included here

The final coding framework comprised 124 codes. The codes were mapped to the intersections between the interview focus areas and the COM-B system and nine summary statements were created, one for each intersection (Table 2). Some codes were allocated to more than one intersection. The three intersections with ‘opportunity’ had the most codes, indicating that factors external to our participants had the biggest impact on their health behaviours. ‘Social connections for health’ was integral to participants’ capability, opportunity and motivation for health. Nine themes and eight sub-themes were derived from the summary statements, with considerable overlap between themes. Themes related to digital health information and supports are reported elsewhere.

**Table 2:** Summary statements and touchpoints (these are explained in more detail below, before being adapted into themes and sub-themes)

	Perceptions of health and health behaviours	Social connections for health	Digital health information and supports^a^
**Capability** for health behaviours	Capability comes from a holistic understanding of health, health information and feeling empowered. **Theme:** Health is multidimensional and a few key health behaviours are valued over all others **Sub-theme:** Health information can empower and disempower health behaviours **Sub-theme:** Everyday life can empower and disempower health behaviours	Social connections and shared experiences are integral to capability. **Theme:** Social connections and shared experiences build knowledge and increase confidence in decision-making. **Sub-theme:** Looking to other sources of information when health professionals are not trusted	Digital health information and supports increase capabilities for health behaviours. **Theme:** Digital health information and support needs to be evidence-based and reputable to increase capability.
**Opportunity** for health behaviours	A range of resources create opportunities for health behaviours. **Theme:** Personal responsibilities prioritised over health behaviours **Sub-theme:** Resources that make health behaviours easier	Social connections are shared experiences are integral to opportunity. **Theme:** Social connections and shared experiences increase opportunity for health behaviours **Sub-theme:** Being listened to shared experiences are important	Digital health information and supports increase opportunities for health behaviours. **Theme:** Digital health information and support needs to be relevant and increase opportunity for social connection **Sub-theme:** Ease of access and convenience are essential **Sub-theme:** Chat-bots and virtual assistants may be useful but they probably will not replace authentic social interactions
**Motivation** for health behaviours	A desire to feel well and live well motivates health behaviours. **Theme:** A range of factors motivate health behaviours. **Sub-theme:** Pregnancy planning motivates behaviour change.	We thrive when we are together. **Theme:** “We’re not designed to be alone… We thrive when we’re together” **Sub-theme:** A range of motivating benefits come from social connections	Digital health information and supports have the potential to increase motivation for health behaviours. **Theme:** New digital health information and support needs to be innovative to meet expectations of their target audience.

^a^Not reported in detail

### Capability

#### Theme

Health is multidimensional and a few key health behaviours are valued over all others.

Contributing to capability, participants had a holistic understanding of health, acknowledging physical, social and emotional dimensions. Nutrition, sleep and exercise were most often raised as important health behaviours in the context of social connectedness and healthy relationships. Participants with children tended to prioritise sleep because a lack of sleep made other health behaviors including a healthy diet and physical activity more challenging. In one of the visual prompt activities, participants identified images of people spending time together, often in the outdoors, as a health behaviour that was good for their physical, mental and emotional health.

*“I think for me, yeah, that, that picture of the two women talking, like healthy relationships is a big part of my personal health as well and my social health.” (P02 Non-intender).*

*“I just think sleep is super, super important for me personally because I have young children and quite often wake up during the night. Good quality sleep, it’s just really important so I can function well the next day.” (P01 Intender).*

*“Getting outside, like it’s the oxygen, but also the change of scenery and being in nature and just being around someone in nature rather than just being inside... It’s good to get out. It just knowing that, cool, in 20 minutes I can get all these benefits and it’ll kind of carry me through most of the day.” (P04 Non-intender).*

#### Sub-theme

Health information can empower and disempower health behaviours.

Participants’ capabilities were developed via an abundance of readily available information from health professionals, online and offline sources and shared experiences. Community advisors and participants identified that health literacy contributed to individuals’ confidence and ability to advocate for oneself; however, too much information was considered overwhelming. Participants felt disempowered by health professionals who did not listen to them, or gave them a textbook answer that did not meet their specific health needs. On the other hand, participants felt empowered by real-life stories from other women who had similar experiences.

*“So, looking for information, I think you can definitely find everything. But sometimes I think it can just be a little bit overwhelming. And, um, maybe there’s a lot of pressure… You feel like knowing so much information can kind of put more pressure on you.” (CO Community Advisor).*

*“I would safely say, I know more about endo* [endometriosis] *than the average doctor. Like I could say that I’ve done hours and hours of research… I’ve had a lot of interactions with old doctors who have, like, belittled my experience or told me I need to go on antidepressants or mood stabilizes or the pain can’t be that bad. Or it’s just a period.” (P03 Non-intender).*

Our community advisors agreed that information in the form of shared experiences was as valuable as traditional sources of health information, if not more valuable.

*“Having that shared experience from somebody who’s gone through it, I can see why women would be crying out for that. Just that idea that someone gets it. Someone’s been through it… It seems that it’s becoming pretty clear in this stage of research that another pamphlet is not going to make much of a difference. Or another factsheet with the information that we may or may not know in the, in the vast world of a lot of information handed to you. If it’s that personal experience that’s going to touch a nerve and make a difference. If that’s what women are identifying with… Do you listen? In a perfect world, I’d say, listen to them and then provide that level of support and shared experience and peer support that they’re craving. Easier said than done though!” (AD – Community Advisor).*

#### Sub-theme

Everyday life can empower and disempower health behaviours.

Negative impacts on participants’ capabilities and health behaviours included chronic conditions such as endometriosis, mental health, stress, being busy and the COVID-19 pandemic. For example, participants reported a range of health behaviours that they believed they were doing well in, including eating healthily and exercising; however, these behaviours were challenged when they were stressed, worried, juggling work and caring duties, or locked down as a result of the COVID-19 pandemic.

*“Like sleep, food, exercise. I mean, when I’m stressed, I know that I don’t seek that out. I don’t eat on time. So I usually skip, I, I like, I keep on skipping meals, which doesn’t help. And, um, even with exercise, I’m just not in the mood to do it, although I know that, okay, this is the time, but I just don’t do it.” (P01 Non-intender).*

*“I really think having a desk job where you’re in meetings a lot and… So you just end up being in the same place a lot, which I don’t think is great. The other thing would be COVID. Like, I used to swim a couple of times a week. But now I’m a bit worried about going swimming cos I’m like, oh, is it a* [safe] *place? I guess now being pregnant too, just being a bit worried about that. But I think, yeah, COVID has certainly had an impact.” (P03 Intender).*

#### Theme

Social connections and shared experiences build knowledge and increase confidence in decision-making.

The importance of social connections to increase participants’ capabilities for health behaviours, including those related to preconception health, was strongly represented in all interviews. Social connections included interactions with health professionals, family, friends, colleagues and communities. Family and friends seemed to have the biggest influence over participants’ health behaviours. Shared experiences were important because, *“When we share our experiences and we share connection with others, or we share our problems or our worries. Then that in and of itself is therapeutic”* (P02 Intender).

Describing support during pregnancy (after a miscarriage and a stillbirth): *“She* [participant’s mother] *would always like say, ‘Hey be positive about it. You’re going to have a good experience’. Um, just, you know, being neutral, but also positive about it. Not bringing up any negative experiences. And so family support was great… Uh, I wanna say they were more supportive and loving than telling me what to do… Yeah. Um, but, um, I think just the love and support was like the biggest thing for me.” (P06 Intender).*

Social connections extended online, with interactions within online communities evolving into trusting relationships that supported participants’ decision-making.

*“PCOS, like, I think I can lecture you on it. It’s not just medicine, but all of the research out there and everything, so I knew what I knew those things already, but I think I needed a different perspective and, uh, people who actually were going through it to share this story… So it was, for me, it was not the information which I was looking for, but more of that connection. Those stories, which I could actually relate to. So they were people who were sharing their own stories and the parts of those stories where things, which I have seen in my life or which I have experienced as well.” (P01 Non-intender).*

*“Um, but I feel like since launching the online community, um, there’s a certain validation that comes with hearing other people, having had the same experiences as you. So, you know, we have a support group on there. Um, people are able to find people who deal with the same issues as them and have someone to talk to.” (P03, Non-intender).*

Simply being listened to had a positive impact on participants’ confidence. Participants felt validated when someone else listened to them, with or without providing advice.

*“I think I like being listened to and then having stuff sort of echoed back to you as well can help you understand how you’re feeling or what’s going on for you.” (P02 Non-intender).*

#### Sub-theme

Looking to other sources of information when health professionals are not trusted.

Social connections included trusting relationships with health professionals and complementary medicine practitioners. If participants did not feel as though health professionals were listening or providing them with the support and advice they wanted, they would seek advice elsewhere.

*“I think people were looking originally to doctors for information… And then when you’re not really satisfied with that, you look to peers and that’s why* [participant’s online community] *has become what it is… It is because so many people are like, okay, well, you might not be a doctor, but you’ve lived this… And we actually trust you more than the doctors.” (P03 Non-intender).*

*“With my painful periods, it was like, ‘Oh, we* [health professional] *can’t help you. You just have that.’ And mainstream medicine had nothing to offer me really. I was like, ‘This can’t be right. There must be something going on here.’ And, um, then I found that these complementary medicines did make a difference and I don’t feel like it would have just resolved itself… I work for a research organization and should be highly sceptical of naturopathy and acupuncture. Um, but I feel like, um, some of these other sources of healthcare have been around for a really long time and we don’t really value those.” (P03 Intender).*

### Opportunity

#### Theme

Personal responsibilities prioritised over health behaviours.

A plethora of external factors impacted on participants’ opportunity for health behaviours. Aspects that participants identified as barriers to health behaviours included work commitments, sedentary jobs, time caring for children and family, stress, lack of support from family, financial health and the COVID-19 pandemic. Participants’ responsibilities seemed to take a higher priority than optimising health behaviours.

*“When you have really young children, babies and things like that, going to the gym is very challenging. So that’s why that’s something that’s not really relevant to me at this stage.” (P01 Intender).*

#### Sub-theme

Resources that make health behaviours easier.

Participants were proactive and resourceful in overcoming barriers to health behaviours. They listed a range of strategies including seeing health professionals or complementary medicine, seeking accountability from friends and family, learning from others’ experiences, and accessing various online health resources (apps, podcasts, social media platforms and online health classes). The COVID-19 pandemic increased participants’ reliance on participation in online health classes such as Pilates and yoga, and Telehealth, instead of face to face interactions.

*“I think as I mentioned, having a regular check in with my psychologist and I do meditation and mindfulness fairly regularly. Acupuncture also provides a kind of weekly input from that point of view because you have your head into the table and you’re meditating.” (P02 Intender).*

*“Just knowing what I am like in terms of accountability and goal setting… Like I can set my own goals, but if I make it, then no one else knows about it, then it doesn’t matter. But if you’re in a group, then I can specify… ‘Here’s something I’m really working on and I’m struggling with it.’ That’s much more likely to bring about change.” (P04 Non-intender).*

*“I started feeling uneasy and that’s when I started going for long walks and I started listening to podcasts. So I listened to a lot of podcasts related to health.” (P01 Non-intender).*

#### Theme

Social connections and shared experiences increase opportunity for health behaviours.

Participants’ identified social connections that contributed to their opportunity for health behaviours within their ‘inner circles’ (i.e. those closest to them, most trusting relationships), their communities and broader environments. Generally, inner circles comprised of family, partners and close friends; communities were family, friends and work colleagues; and the broader environment comprised of acquaintances, health professionals and governments.

*“So I’ve got sort of a group of close friends… And then, yeah, I guess our family, I hesitate like, you know, obviously your family are your inner circle, but sometimes like they’re not necessarily the people that I would share that the deepest darkest beings with all of the time..*. *I’m very close with a number of my colleagues and, you know, some of them probably at times cross over into that inner circle sort of friendship group.” (P02 Intender).*

In relation to participant’s broader environment: *“I’ve got two GP clinics I go to wherever I can get an appointment. Um, so those people… Shopkeepers… And then other parents that you might see them at the cafe they own, and you see them at after school care and footy or Auskick or something like that and you have a chat.” (P04 Non-intender).*

Social connections seemed to have the biggest impact on participants’ opportunity for positive health behaviours (more so than ‘perceptions of health and health behaviours’ or ‘digital health information and support’). Social connections for health impacted all aspects of the COM-B system, including knowledge, health literacy, feelings of confidence and desire to adopt health behaviours. Community advisors agreed with the importance of social connections to optimise health behaviours.

*“Um, why are they* [social connections] *important? I guess just to feel like you’re part of a group. Like it’s nice to feel included and to have people that you can rely on and people that rely on you... So I guess like from some people I’m getting love and time and doing activities together. Yeah, having fun… Um, and then others, like I said, I’m getting like mentoring… I guess other people that are living their life a certain way that I think, yeah, that’s how I also want to be living my life… Different perspectives on things and broaden my world view a little bit… And even just that physical yeah, physical touch. I’ll say like being able to get a hug from someone.” (P04 Intender).*

*“I’d have to agree with what a lot of the women are feeding back through that social connectedness… Is wildly important. Um, and so we can tie that in with health promotion. I think you’re going to get some positive results. And what that actually looks like is something that wish I could answer for you.” (AD, Community Advisor).*

#### Sub-theme

Being listened to shared experiences are important.

Social connections provided practical and emotional support for health behaviours. As mentioned previously, the most valued supports were being listened to and shared experiences. Inner circles were the most relied on for these supports and participants were generally satisfied with the support they were receiving.

*“I think one example is when, um, when I was going through quite a busy period at work, um, I was able to just touch base with my friend and talk to her about how I was feeling about all about, um, yeah. And what, what that sort of meant for me and meant for my energy levels. And, um, and she didn’t necessarily offer any thing or any advice she just asked questions and was interested… Um, and just listened, I guess.” (P02 Non-intender).*

*“I think my family, particularly with the pregnancy losses and going through IVF, like we were really open in sharing what was going on. I know not everyone has that situation or feels like that’s what they want to do, but I felt like that was really important not to just hold, hold our story… And kind of to share our stories with other people. I think that was an important coping strategy for us through the last few years.” (P03 Intender).*

### Motivation

#### Theme

A range of factors motivate health behaviours.

Feeling well and living life to the full motivated participants to adopt and maintain health behaviours. Weight-loss or achieving a healthy weight rarely came up as a motivator for behaviour change. On the other hand, being in nature motivated most participants to exercise, feeling well motivated participants to nourish well, and mental health motivated participants to prioritise rest and relaxation.

*“Something was not working. And that’s when, that’s when I started walking. Like, just stepping out of the home and just making the most of that one hour of exercise. Because I used to think that exercise was like, going to the gym…. My focus was always losing weight, but that should not be your focus. Your focus should be feeling good. So that’s what walking did for me. So, it was making me feel good. Like, that one hour was for me. I wasn’t talking to others. I was not thinking anything else… I just, um, took off my headphones and then just walked and admired the leaves, the people, the dogs. Just nature. That’s it. Yeah.” (P01 Non-intender).*

*“It’s good to get outside. Just to get some exercise to have a physical benefit, and mental, or all the other benefits… Like, I found a few bike paths in our area… And that has been so good because the bike track is along like a waterway kind of creek type thing. There’s lots of trees. It’s just a really nice. It’s not just about the physical element. It’s about being out in nature, just me and my thoughts, having that space. I just let my thoughts go wherever and not have to focus on anything else then. Yeah. Um, yeah, so that that’s been really good.” (P04 Non-intender).*

One participant’s goal was meal planning for the week (P04 Non-intender), another participant’s goal was to walk on most days of the week (P01 Non-intender), and another participant’s goal was to go to bed 30 minutes earlier (P05 Intender). Experiencing the positive physical and mental health outcomes after a health behaviour (e.g., going for a long walk) motivated participants to continue the health behaviour.

*“Seeing things improve really motivates me… I felt really nauseous this morning and I said, ‘I’ve got to go for a walk.’ I said, ‘Come on, come on, come on, we’ve got to go.’ And now I feel a completely different person... So I think, you know, if I can get that sort of instant gratification, that’s really motivating for me. And then I’m like, ‘I’ve just got to do this every day I can,’ so that I feel better. (P02 Intender).*

In ‘intender’ participants, preparing for pregnancy motivated behaviour change. Those who had experience with IVF gave detailed descriptions of their lifestyle changes, for them and their partners. These lifestyle changes included nutrition, exercise, shifting to low chemical cleaning and skincare products, and seeking complementary health care such as naturopaths and acupuncture. Partners’ lifestyle changes involved improved nutrition and decreased alcohol consumption.

*“Obviously wanting to be a parent is a strong motivator.” (P03 Intender).*

*“My attitude* [about health] *now is a bit more blaze, but when I was planning to get pregnant with* [son] *it was like really a big deal to me… I don’t want to do anything that’s gonna jeopardize my child’s, you know, health or you know… I wanted do everything possible and I think I did all the, you know, um, you know, no drinking and eating rubbish and all that for probably a couple of months before we started trying and all that sort of thing. I think I even bought like, you know, herbs and bits and pieces and stuff because I researched them… It’s like, you know, you’re carrying this precious thing and precious things need to be looked after… If you had a garden, like you’re not going to plant a plant in crap soil or whatever, you going to plant in nice soil and look after it and nurture it and things like that.” (P06 Non-intender).*

The possibility of a future pregnancy was not a motivator for lifestyle change for participants who were not planning a pregnancy.

*“And they* [preconception health behaviours] *are usually things that I haven’t thought about before, because I haven’t been pregnant before and I haven’t, I guess, gotten close to becoming pregnant before either. So it’s not something that I’ve started to consider.” (P02 Non-intender).*

#### Theme

“We’re not designed to be alone… We thrive when we’re together.”

Participants were highly motivated to connect with others. Social connections were considered crucial for health and wellbeing with comments such as, *“Humans are meant to connect socially. It’s just part of how we evolve,”* (P01 Intender); and, *“We’re not designed to be alone… We thrive when we’re together,”* (P02 Intender).

*“Yeah, so getting outside with family… So getting exercise, being out in the fresh air and nature, the social aspect of spending time together and having connection. Um, yeah. That’s what like, I really do try to, yeah. Um, yeah, I, I value that as an activity.” (P04 Non-intender).*

*“I feel like I’m at my best, if I’m spending lots of time with women. Um, and because the things that you talk about are just different. Like, as much as you love your partner, like your husband or boyfriend or whatever, there’s just certain things that you just, you just can’t, you can just say around other women… they were going to just going to have a better understanding of it. And so going for like a long three hour, four hour ranting walk… I just, I always feel like invigorated afterwards.” (P05 Non-intender).*

#### Sub-theme

A range of motivating benefits come from social connections.

Participants described how social connections enriched their lives, created a sense of purpose and belonging and motivated them in healthy decision-making. Participants discovered more about themselves and learned from other people’s knowledge and lived experience.

*“You feel supported, you feel understood your feelings are validated. Um, I think, you know, it gives you the energy to keep doing what you’re doing and there’s other people just like you, not the same but are going through similar things.” (P01 Intender).*

*“They* [social connections] *enable us to discover and explore the side of ourselves, um, or at least develop, um, a deeper sense of purpose. And yeah, I think this sense of purpose that’s most important… And being able to sort of anchor yourself amongst sort of other people and knowing where you are and how other people have gone through similar things… Like it’s knowing that it’s normal and hearing how other people have overcome their challenges.” (P05 Intender).*

## Discussion

This research gathered pregnancy ‘intender’ and ‘non-intender’ women’s views of their health and preferred supports to optimise health behaviours in the preconception period. Over several months and multiple in-depth interviews, researchers were able to gather deep insights into women’s lived experiences and their reliance on social connections for health. There were subtle differences between intender and non-intender women, the most notable being potential pregnancy as a motivator for behaviour change in intender women. Overall, participants were motivated to achieve the highest level of health and wellbeing attainable for them, regardless of pregnancy intention. Everyday life, including illness, being busy, caring for others and the COVID-19 pandemic got in the way of maintaining health behaviours. However, participants were resourceful as they used social connections to increase their knowledge, opportunity and motivation to adopt and maintain health behaviours that were important to them.

Our participants had a holistic view of health, acknowledging the importance of physical, mental and social dimensions and describing how one dimension may impact on another. Nutrition, physical activity and sleep were prioritised above other individual health behaviours, reflecting findings in other research that has explored health behaviours that are important to preconception women [16, 30]. Weight or physical appearance were seldom raised as important aspects of health or motivators for health behaviours by our participants, contrasting with how the media portrays optimal health alongside having a ‘perfect’ body [31, 32]. Our participants and community advisors were wary of unrealistic expectations promoted by the media, preferring practical and achievable strategies to optimise aspects of health most important to them. Interventions that target health behaviours in the preconception period should consider women’s preferences for holistic health and wellness messaging [32], and contain clear and realistic advice around aspects of health considered most important, namely nutrition, physical activity and sleep. Consideration should also be given to women’s reliance on social connections that support these behaviours.

Social connections for health was raised throughout all the interviews, having a strong influence over participants’ health, health behaviours and motivations for health. Participants actively sought support from others, either by being listened to, or from learning from others’ shared experiences (or experiences in common). Social connections that provided the most meaningful support for our participants came from those closest to them (e.g., family, partners, close friends). Being listened to was a key feature of this support, consistent with previous research that describes the power of listening in close relationships [33]. The importance of social connections for health was further described by Small et al., [34] who explained that supports valued by women were those that provided non-judgmental companionship, a listening ear, and reassurance. Being listened to develops close emotional connections and creates a sense of being valued, cared for, and empowered [33]. Furthermore, vocalising thoughts or experiences supports individuals to reflect and increase self-awareness of capabilities, opportunities and motivations for health behaviours [33]. Shared or common experiences have positive emotional benefits for individuals and groups, providing reassurance, learning opportunities and guidance in decision-making [35]. Overall, this research highlights how the combination of being listened to and learning from the experiences of others are powerful enablers of health and health behaviours.

Some social connections described by our participants started through online communities before evolving into close friendships. Connections as a result of shared or common experiences were sought online, mainly through Facebook, Instagram and Podcasts. *‘For me, it was not the information I was looking for, but more of that connection. Uh, those stories, which I could actually relate to’* (P01 Non-intender). Participants felt validated hearing others’ similar experiences, albeit online. Consequently, trusting and close relationships were developed. Smith-Merry et al. [36] also described how the desire for shared experiences was a strong motivator to engage with online forums for mental health, and how shared experience could help to normalise one’s experience. In our study, online communities created around shared or common experiences gave participants a sense of belonging and alleviated isolation. The provision of information and advice was secondary to social connections and learning from others’ lived experiences. This is a challenging consideration for developers of behaviour change interventions, where imparting evidence-based knowledge or education is viewed as a key aspect of supporting individuals to move from one stage of change to another [37]. While evidence-based education is important, our data point to the value the real-life experiences of others to support women to move from contemplation to preparation, action and maintenance.

Social connections or online communities were sought when participants were not satisfied with the information or support they were receiving from their health professionals. Participants often turned to peers with, *‘You might not be a doctor, but you’ve lived this…And we actually trust you more than the doctors,’* (P03 Non-intender). Again, this reflects the trust found in shared or common experiences and meaningful social connections [38, 39]. Trust in a health professional can affect patient satisfaction with care [38], have positive effects on patients’ engagement with advice, and ultimately improve patient outcomes [40]. Building trusting connections is an important consideration for health professionals [41], and is therefore an important consideration in the development of behaviour change interventions. Our research highlighted how target audiences will be more receptive to messages from sources where they feel there is a connection and trust, and a shared or common experience [42].

A potential pregnancy is a strong motivator for behaviour change in women who are trying to concieve [28]. This was generally seen in our participants, and was the biggest difference between our non-intender and intender groups. Interestingly, our data suggested that for those intending to become pregnant within approximately 2 years, future pregnancy was not yet a motivator for behaviour change. This finding was reflected in our intender participants and in our community advisory committee, regardless of parity. It seemed that if pregnancy was not a shorter-term goal (e.g., intention to conceive in the next 3 months or so) then it was not a motivator. This important finding requires further exploration. One or two years may not be long enough for some woman to address sub-optimal preconception health behaviours and achieve optimal preconception health. For example, it may take a woman with a body mass index of 40 kg/m^2^ more than 2 years to achieve an optimal preconception weight. Therefore, public health messages need to support individual behaviour change interventions by increasing awareness that lifestyle behaviours more than just a few months before conception may still impact on fertility and pregnancy outcomes [1, 2]. Those developing public health messages and behaviour change interventions that promote preconception health need to be mindful that not all women want to have children, that women’s needs and preferences change across the lifecourse, and that all women, regardless of pregnancy intention, have a holistic view of health and value their overall health and wellbeing and they gain informational and emotional support in the context of social connection.

### Strengths and limitations

Our community advisors were a study strength as they provided feedback on data collection, data analyses, results and potential directions for the next phase of this research. There was equal representation from intender and non-intender participants and from the first in-depth interview, 100 % participant retention. Researchers and participants were able to develop rapport and trust as they met regularly over the period of 3 months, albeit online. Multiple interviews over time created opportunities to delve deeply into participants’ lived experiences of their health behaviours and desired supports for health and wellbeing. Additionally, regular meetings with the research team and ongoing data analyses throughout the engagement period with participants and community advisors enabled the interviews to be tailored to individual participants.

The COVID-19 pandemic meant that our participant interactions and community advisory committee meetings were online. Zoom facilitated face-to-face interactions and also made it possible to engage with participants from regional Victoria who may not have otherwise volunteered to participate. However, relationships built via in person interactions were not established. All of our participants had experienced the isolation of several lockdowns to slow the spread of COVID-19 over the past 18 months. This may have influenced the results that emphasized how participants valued and relied on social connections.

A limitation was that the median age of participants was 31.5 years (IQR 8). The views of women aged 18-26 years were not represented in our participant sample. Our community advisors, three aged 23 years, filled this gap to a degree. Participants and community advisors who could not speak English were excluded, limiting the generalizability of this research to women who can speak English. Other aspects that may limit the generalizability of this research are that many of our participants were recruited from women’s health groups in the community and were likely to have had a specific interest in women’s health issues, and most of our participants were mostly university educated. Further work is required to understand how to develop interventions that meet the needs of women in the community who experience socio-economic disadvantage and isolation.

## Conclusions

This study provided insights into preconception women’s understandings of health and health behaviours, and what supports they value to optimise health behaviours. Women had a holistic view of health and were generally motivated to achieve optimal health and wellbeing, regardless of their pregnancy intentions. Women proactively sought information from a wide range of existing sources but a barrier was feeling as though they were not being listened to. Social connections for health, including being listened to and shared experiences, were most highly valued supports for health behaviours. Future interventions should consider women’s needs and preferences for social connections over yet more evidence-based information, and leverage off existing online platforms that facilitate these connections.

## Supplementary Information

**Additional file 1: Supplementary file 1.** The development of themes and sub-themes from codes*

## Data Availability

The data generated and analysed during the current study are not publicly available due to this project being ongoing but are available from the corresponding author on reasonable request.

## Abbreviations

COM-B system: Capabilities, opportunities, motivation – behaviours
IQR: Interquartile range
