# Supplementary material for: Empowerment for behaviour change through social connections: a qualitative exploration of women’s preferences in preconception health promotion in the state of Victoria, Australia
Source: BMC Public Health. 2022 Aug 30;22:1642. doi: 10.1186/s12889-022-14028-5 (PMC9425810; doi:10.1186/s12889-022-14028-5)
Supplement: Supplementary file 1 — Additional file 1: Supplementary file 1. The development of themes and sub-themes from codes* [file 12889_2022_14028_MOESM1_ESM.docx]

**Supplementary File 1:** The development of themes and sub-themes from codes*

|  | **Women’s perceptions of health and their lifestyle behaviours** | **Social connections for health** | **Digital health information and supports** |
| --- | --- | --- | --- |
| **Capability** (psychological and physical capacity, including knowledge and skills) | Advice – 21  Assumptions of health – 5   - Relevant information (CAC_Codes specific to this) – 6   Chronic illness or pain – 30  Doing well for health – 19  Exercise – 99  Health advocacy_empowerment - 17  Health literacy - 19  Health_Holistic -26  Important for health 30  Information_Lots - 2  Information_No more - 16  Information_Sources not online - 11  Mental health - 73   - Stress (Mental health) - 13   Multitasking - 23  Nutrition - 117  Routine_Planning - 18  Rest_Relaxation – 39  Seeing HPs - 46  Sleep - 51 | Chronic illness or pain - 30  Seeing HPs - 46  Shared experience_stories_views - 73   - Importance of social connections (social connections) - 31 | Desirable IT features   - Evidence-based (Desriable IT features) - 9 - Factsheets (Desriable IT features) - 5 - Moderated (Desriable IT features) – 12 - Notification (Desriable IT features) - 5 - PC specific wants - 12 - Survey at start to tailor advice_or tailored info (Desriable IT features) - 9 - Tracking (Desriable IT features) - 11   Health literacy - 19  Information_Sources online -15   - Apps general (IT) - 15 - Devices used (IT) - 21 - Ways devices used for health (IT) - 60   Online for health   - Apps (online for health) - 34 - Online classes (online for health) - 4 - Podcasts (online for health) - 20 - Search methods for health issue (online for health) - 21 - Websites (online for health) - 3 |
| **Opportunity**  (factors external to an individual that make a behaviour possible, including physical environment and cultural milieu) | Accountability – 14  Advice – 21  Balance for health - 16  Barriers – 54  Caring for people as part of wellbeing – 7  Children_Impact on life or health – 23  COVID-19 - 52   - IT fatigue (COVID) - 5   Financial health - 12  Important for health 30   - Community_forum (online for health) - 15 - Facebook (online for health) - 27 - Instagram (online for health) - 24 - Name of a health app used (online for health) - 23 - Barriers (preconception) - 4   Preconception_Supports – 37  Seeing HPs - 46  Shared experience_stories_views - 73  Support_Not getting  Week_Work_Weekend - 74  Online for health   - Apps (online for health) - 34 - Online classes (online for health) - 4 - Podcasts (online for health) - 20 - Search methods for health issue (online for health) - 21 - Websites (online for health) - 3 - Preconception changes (preconception) - 23 - Information (Preconception_Supports) - 20 | Accountability – 14  Advice – 21  Being heard_Listened to -17  Barriers to social connection – 12   - Rapport participants (CAC_Codes specific to this) - 20   Caring for people as part of wellbeing – 7   - Online groups (Desriable IT features)   Culture_Diversity - 5  Makes you feel connected - 11   - Community_forum (online for health) - 15 - Social media_general (online for health) - 42   Preconception_Supports – 37  Shared experience_stories_views - 73  Social connections   - Broader environment (social connections) -25 - Community (social connections) - 34 - Inner circle (social connections) - 41 - Relationships_Family (social connections) - 51 - Relationships_Friends (social connections) - 55 - Relationships_Other (social connections) - 19 - Relationships_Partner (social connections) - 24   Support_Not getting  Trust – 4  Busy – 23   - Social determinants (CAC_Codes specific to this) - 8   Supports for health   - Emotional support (supports for health) - 47 - Physical support (supports for health) - 56 | Advice – 21   - Free or right price (Desriable IT features) - 6 - Links to local services (Desriable IT features) - 2 - Online groups (Desriable IT features) - Safety net ie.. Lifeline (Desriable IT features) - 1 - Shared experiences (Desriable IT features) - 16 - Useful or not useful for health (IT) - 16 - Virtual assistants.chat bots (IT) - 19 - Social media_general (online for health) - 42 - Connecting online (social connections) - 42 - Devices used (IT) - 21   Online community   - Don’t want from online community (Online community) – 18 - Engagement (Online community) – 17 - Want-expect from online community (Online community) – 14 - Community_forum (online for health) - 15 - Facebook (online for health) - 27 - Instagram (online for health) - 24 - Name of a health app used (online for health) - 23 - Access to info when needed (CAC_Codes specific to this) – 6   Online for health   - Apps (online for health) - 34 - Online classes (online for health) - 4 - Podcasts (online for health) - 20 - Search methods for health issue (online for health) - 21 - Websites (online for health) - 3 |
| **Motivation**  (brain processes that energise and direct behaviour, including goal-setting, habits, emotional responses, informed decision-making) | COVID - 52   - IT fatigue (COVID) - 5   IVF - 11  Motivation -23  Outdoors_Nature - 34   - Preconception lifestyle important (preconception) - 14   Rest_Relaxation - 32   - Emotional support (supports for health) - 47   Miscarriage – 8  Realistic expectations - 8  Weight - 10 | - IT fatigue (COVID) - 5 - Importance of social connections (social connections) - 31 | - IT fatigue (COVID) - 5   Desirable IT features - 26   - Clear purpose (Desriable IT features) - 7 - Engaging.visual.user experience (Desriable IT features) – 9 - Honesty_Trust (Desriable IT features) - 7 - Human element (Desriable IT features) - 11 - Platform (Desriable IT features) - 2 - Privacy (Desriable IT features) - 1 - Variety (Desriable IT features) - 14 - Frustrations of online resources (online for health) - 12 |

* Considerable overlap between codes, some appearing in more than one theme.
